# Impact of a Mobile Health Application on Medication Adherence, Thyroid Function, and Quality of Life in Patients with Hypothyroidism: A Prospective Comparative Study

**DOI:** 10.12669/pjms.42.9.14616

**Published:** 2026-09

**Authors:** Saima Askari, Eraj Abbas, Azra Shaheen, Mahwish Bano

**Affiliations:** 1 Dr. Saima Askari, MBBS, FCPS Medicine, FCPS Endocrinology, CHPE, Assistant Professor & Consultant Endocrinologist, Baqai Institute of Diabetology & Endocrinology, Baqai Medical University, Karachi, Pakistan; 2 Dr. Eraj Abbas, Assistant Professor, Department of Biochemistry, Baqai Medical University, Karachi, Pakistan; 3 Dr. Azra Shaheen, PhD Clinical Psychology, Associate Professor, Department of Psychiatry and behavior science, Baqai Medical University, Karachi, Pakistan; 4 Dr. Mahwish Bano, BDS, MPH, CHPE Assistant Professor, Community Dentistry Department, Baqai Dental College, Baqai Medical University, Karachi, Pakistan

**Keywords:** Mobile Health Application, Hypothyroidism Management, Treatment Adherence, Symptoms Improvement

## Abstract

**Background & Objective:**

Effective hypothyroidism management requires consistent daily levothyroxine therapy; poor adherence causes fluctuating Thyroid Stimulating Hormone (TSH), persistent symptoms, and long-term complications. To evaluate the impact of a mobile application on symptom improvement and clinical outcomes through improved treatment adherence among patients with hypothyroidism.

**Methodology:**

The study was conducted at the Outpatient Department of Diabetes & Endocrine center, Karachi over a duration of 10 months from 1^st^ January 2025 to 31^st^ October 2025. A total of 60 participants were included and divided into two groups: Group-1 (Control), did not use the mobile application, and Group-2 (Test), which used the mobile application. Patients aged 18 to 60 years with a confirmed diagnosis of hypothyroidism requiring ongoing levothyroxine therapy were included. Patients with hyperthyroidism, thyroid cancer, thyroid nodules or severe comorbidities were excluded. Symptoms (weight gain, cold intolerance, increased sleepiness, menstrual irregularity), TSH levels and Quality of Life Scale (WHOQOL), were assessed at baseline, after two and six months in both groups.

**Results:**

Mobile app users showed lower mean TSH levels (4.0 ± 2.10 mIU/L) than non-users (6.78 ± 2.78 mIU/L; p < 0.001) and higher quality-of-life scores (82.43 ± 18.25 vs. 72.10 ± 21.64; p<0.01) at six months. The symptoms improvements were more pronounced in application users as compared to non- users especially in menstrual irregularity (40.0% vs. 70.0%; p < 0.001), fatigue (50.0% vs. 66.6%; p = 0.01) and weight gain (16.6% vs. 60%, p<0.01).

**Conclusion:**

The study revealed that integrating mobile health applications into routine hypothyroidism care can substantially enhance treatment outcomes and provide a simple and effective strategy for medication adherence.

## INTRODUCTION

Optimal management of hypothyroidism relies on sustained adherence to daily administration of thyroxine (T4), to prevent adverse health outcomes.^1^,^2^ Hypothyroidism is a chronic endocrine disorder characterized by the insufficient production of thyroid hormones, typically necessitates lifelong treatment with levothyroxine.^3^,^4^ Inadequate adherence to this regimen can result in persistent clinical symptoms, progression of disease, and deterioration in patients quality of life.^5^ Medication adherence, however, is a multidimensional concept encompassing timely drug intake, compliance with follow-up visits, and commitment to lifestyle recommendations provided by healthcare professionals.^6^ Multiple factors contribute to suboptimal adherence, including forgetfulness, limited awareness of importance of consistent medication use, complexity of dosing schedules and the burden of sustained long-term therapy.^7^-^9^

The advent of digital health technologies, particularly mobile applications, offer promising solutions to enhance medication adherence. These apps can provide patients with daily reminders, track their medication intake and offer educational resources.^10^ Therefore, it can be hypothesized that the integration of these tools (like Medisafe® application which is freely available) into the management of hypothyroidism may enhance medication adherence, stabilize TSH levels, and ultimately improve the quality of life among patients with hypothyroidism. Although, there is growing evidence that a higher compliance is observed in patients of non-communicable diseases who had been using digital application, no local data is available especially in people with hypothyroidism in this regard.^11^ In low and middle income countries (LMICs) where medication adherence is challenging due to patient burden, lack of education and access to health care, use of mobile health applications may provide a low cost and user friendly method to improve long term disease outcomes. Therefore, the current study aimed to evaluate the impact of a mobile application on symptom improvement, thyroid stimulating hormone level and Quality of life through enhanced treatment adherence among patients with hypothyroidism.

## METHODOLOGY

The study was conducted at the Outpatient Department of Baqai Institute of Diabetology & Endocrinology (BIDE), Baqai Medical University, Karachi, Pakistan. This prospective, comparative, interventional study was carried out over a duration of 10 months from 1 January 2025 to 31 October 2025.

### Ethical approval:

The study commenced after obtaining approval from the Institutional Review and Ethics Board (IREB) letter no 12-2024/017/(FREC-FM/11-2024/06; dated December 11, 2024) of Baqai Medical University. An informed written consent was obtained from all patients prior to their inclusion in the study.

### Selection of participants and sample size:

Participants were recruited consecutively from the endocrine outpatient clinic of BIDE through non-probability consecutive sampling. This was a prospective, non-randomized interventional study. After receiving information about the study and the Medisafe® mobile application, participants who agreed to use the Medisafe® application were allocated to the intervention group, whereas those who declined continued routine standard care and were assigned to the control group. Allocation concealment and blinding were not performed.

The sample size was calculated using G Power software. The proportion formula for two independent samples was applied as per a previous study.^12^ The minimum sample size calculated was n = 60, with 30 participants in each group.

### Inclusion criteria:

The inclusion criteria comprised participants with a confirmed diagnosis of hypothyroidism who required ongoing levothyroxine (T4) therapy, aged between 18 to 60 years, and who owned and used a smartphone capable of running the Mobile Application (Medisafe®), ensuring their ability to engage with the mobile app intervention.

### Study groups:

Participants were divided into two groups:

### Control Group:

Received routine standard clinical care without the use of the mobile application.

### Intervention Group:

Received routine clinical care together with the Medisafe® mobile application.

**Medisafe**® is a smartphone-based medication management application designed to enhance medication adherence by providing personalized medication reminders, dose scheduling, adherence monitoring and medication related notifications. This application was selected as it is widely available, user-friendly and free of cost, making it a practical tool for improving long-term medication adherence in patients with hypothyroidism.

### Exclusion criteria:

Patients with hyperthyroidism, thyroid cancer, thyroid nodules that could influence TSH levels, uncontrolled diabetes, severe cardiovascular disease, renal failure, or diagnosed psychiatric disorders were excluded.

### Study Procedure:

Participants in the intervention group-downloaded the Medisafe® application onto their smartphones. They were educated and guided on how to use the application by principal investigator. After setting up a profile, participants uploaded their medication schedule (medication name, medication appearance, and dosage). Upon completion, participants began receiving customized daily reminders to take their tablet/s. The reminders were standardized in the interventional group throughout the study period

Levothyroxine dosage was adjusted according to American Thyroid Association (ATA) Guidelines. Baseline and subsequent dosage were made during follow up visits based on the serum TSH levels.^13^ The clinical parameters assessed included hypothyroidism related symptoms (fatigue, weight gain, cold intolerance, constipation, excessive sleepiness and menstrual irregularities) and biochemically assessed serum TSH levels. All study data including demographic characteristics, hypothyroid related symptoms and clinical assessments were conducted in person during scheduled Outpatient Department visits by the Principal Investigator and co-investigators. A self-designed structured questionnaire, developed according to the study objectives, was used to collect demographic information, clinical characteristics, hypothyroidism-related symptoms, and follow-up data. Baseline assessments were carried out before the initiation of Medisafe® use, and recorded after two months and six months of usage, in both the control and intervention groups.

### Outcome measures:

The primary outcome of the study was improvement in thyroid function, assessed by change in serum TSH levels from baseline to six months.

The secondary outcomes included improvement in hypothyroidism-related symptoms (fatigue, weight gain, cold intolerance, constipation, excessive sleepiness and menstrual irregularities), quality of life and medication adherence. Symptoms improvement was defined as reduction in frequency or presence compared to baseline.

Standard laboratory protocols were followed for the assessment of TSH levels. Serum TSH levels were measured using a Chemiluminescent Immunoassay (CLIA) according to laboratory protocols. TSH values were reported in mIU/L with TSH ranging between 0.4 - 4.2 mIU/L considered normal. The quality of life of study participants was assessed using the World Health Organization Quality of Life (WHOQOL) scale at baseline, two months and six months in both study groups.

Levothyroxine adherence was estimated from participants’ self-reported adherence during follow-up visits and was interpreted in conjunction with changes in serum TSH levels and improvement in hypothyroidism-related symptoms.

### Statistical Analysis:

Data were analyzed using SPSS version 23.0. An independent t-test, paired t test and Chi Square test were applied for comparison of clinical parameters and quality of life between the two groups. A p-value of less than 0.05 was considered statistically significant.

## RESULTS

In this study, a total of 60 participants were included and equally divided into two groups, app users and non-users. At baseline, both groups, mobile app users and non-users were comparable in their demographic and clinical characteristics. The mean age of study participants was 33.37±8.60 in mobile app users while in non-users it was 33.17±5.99 while all of the participants were females in both groups as shown in Table-I.

**Table-I:** Comparison of Clinical Outcomes Between Mobile App Users and Non-Users at Baseline, 2 Months, and 6 Months.

	At Baseline			At 2 months			At 6 months		
Variables	Mobile App Users	Mobile App Non- Users	p-value	Mobile App Users	Mobile App Non- Users	p value	Mobile App Users	Mobile App Non- Users	p value
	Mean ± SD			Mean ± SD			Mean ± SD		
Age	33.37±8.60	33.17±5.99	0.91						
	*Frequency (%)*			*Frequency (%)*			*Frequency (%)*		
Gender (Female)	30(100%)	30(100%)							
***Fatigue***									
Yes (%)	22(73.3)	20(66.6)	0.07	15(50.0)	20(66.6)	0.01	10(33.3)	20(66.6)	<0.001
No (%)	08(26.7)	10(33.3)		15(50.0)	10(33.3)		20(66.6)	10(33.3)	
***Cold intolerance***									
Yes (%)	20(66.6)	21(70.0)	0.87	17 (56.6)	21 (70.0)	0.01	15 (50.0)	23 (76.6)	0.01
No (%)	10(33.3)	09(30.0)		13 (43.3)	11 (30.0)		15 (50.0)	07 (23.3)	
***Weight gain***									
Yes (%)	16(53.3)	15(50.0)	0.67	12(40.0)	17(56.6)	0.004	05(16.6)	18(60.0)	0.01
No (%)	14(46.6)	15(50.0)		18(60.0)	15(43.4)		25(83.3)	12(40.0)	
***Constipation***									
Yes (%)	14(46.6%)	12(40.0)	0.96	12(40.0)	16(53.0)	0.09	10(33.3)	16(53.0)	0.002
No (%)	16(53.3%)	18(60.0)		18(60.0)	14(46.6)		20(66.6)	14(46.6)	
***Excessive sleepiness***									
Yes (%)	10 (33.3)	14 (46.6)	0.08	5 (16.6)	14 (46.6)	0.01	5 (16.6)	14 (46.6)	0.01
No (%)	20(66.6)	16 (53.3)		25(83.4)	16 (53.3)		25(83.4)	16 (53.3)	
***Menstrual irregularities***									
Yes (%)	22(73.3)	25 (83.3)	0.89	12 (40.0)	21 (70.0)	<0.001	9 (30.0)	20 (66.6)	<0.001
No (%)	08(26.6)	05 (16.6)		18(60.0)	09 (30.0)		21(70.0)	10 (33.3)	

Data are presented as mean ± standard deviation (SD) or n (%).

Hypothyroid related symptoms improved significantly in mobile app users than non-users over the study duration. At two months period, app users reported significantly lower frequencies in fatigue (50.0% vs. 66.6%, *p* = 0.01), weight gain (40.0% vs. 56.6%, *p* = 0.004), excessive sleepiness (16.6% vs. 46.6%, *p* = 0.01) and menstrual cycle irregularities (40.0% vs. 70.0%, *p* < 0.001) relative to non-users. Similarly at six month follow up, comparative to non-users, mobile app users demonstrated notably reduced symptom frequencies in fatigue (33.3% vs 66.6%, *p*<0.001), weight gain (16.6% vs 60.0%, *p* = 0.01), excessive sleepiness (16.6% vs 46.6%; *p* = 0.01) and menstrual cycle irregularities (30.0% vs 66.6%, *p* < 0.001). Detailed changes in frequencies of all hypothyroid related symptoms over the study period are mentioned in Table-I.

Thyroid dysfunction reflected by the TSH level (6.5 ±1.69 vs 6.3 ± 2.4, p=0.95) showed no significant baseline differences between the two groups, confirming that both cohorts were comparable prior to the intervention. Patients using the mobile application for medication reminders showed better thyroid function control (*p* < 0.001) over the study duration as shown in Fig.1.

**Fig.1:**
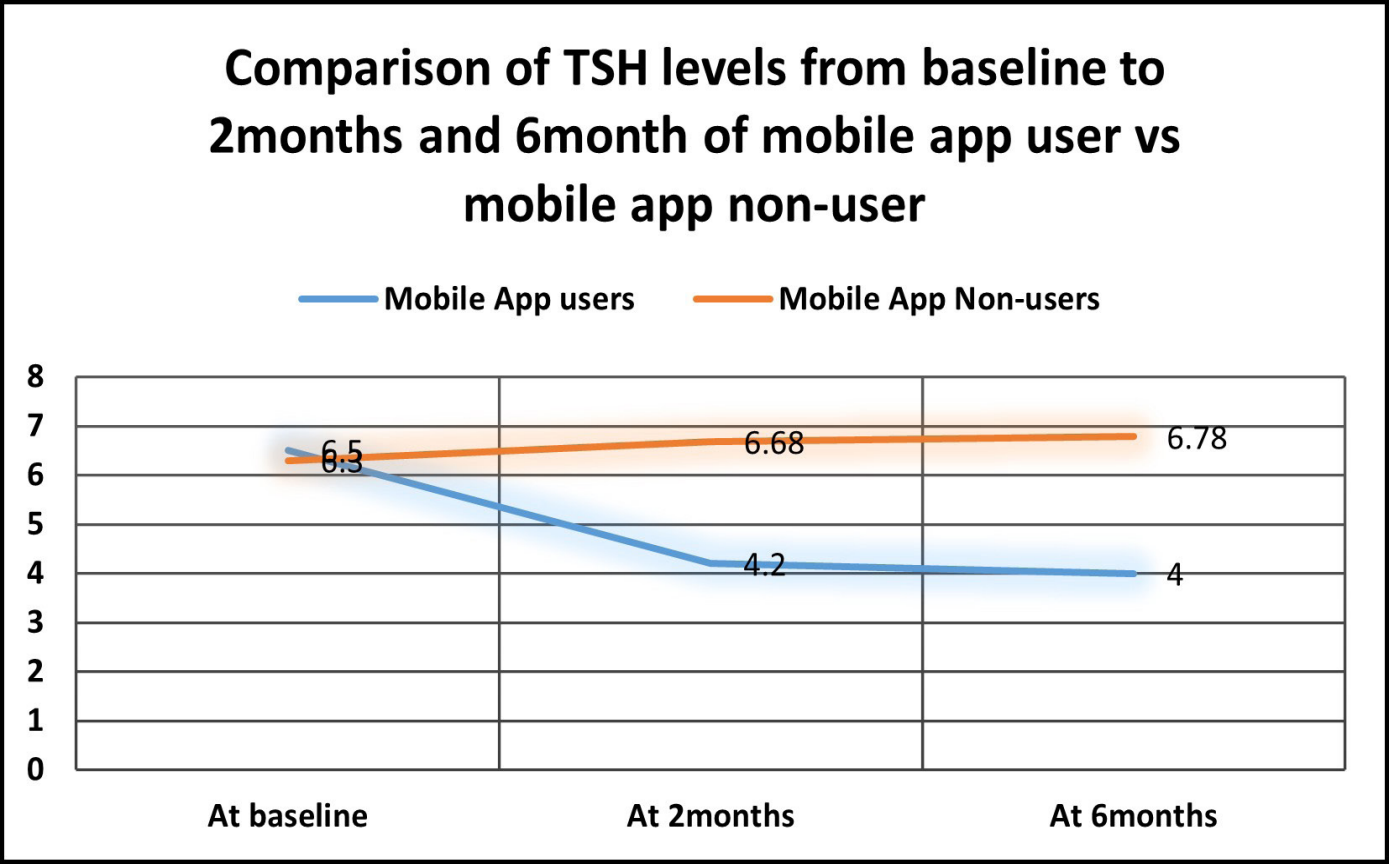
Comparison of Serum TSH Levels (mIU/L) at Baseline, 2 Months, and 6 Months Between Mobile App Users and Non-Users.

A statistically significant improvement in QOL scores over time in the intervention groups compared to controls among Mobile App users and Mobile App non-users patients with hypothyroidism (p<0.01) is shown in Fig.2

**Fig.2:**
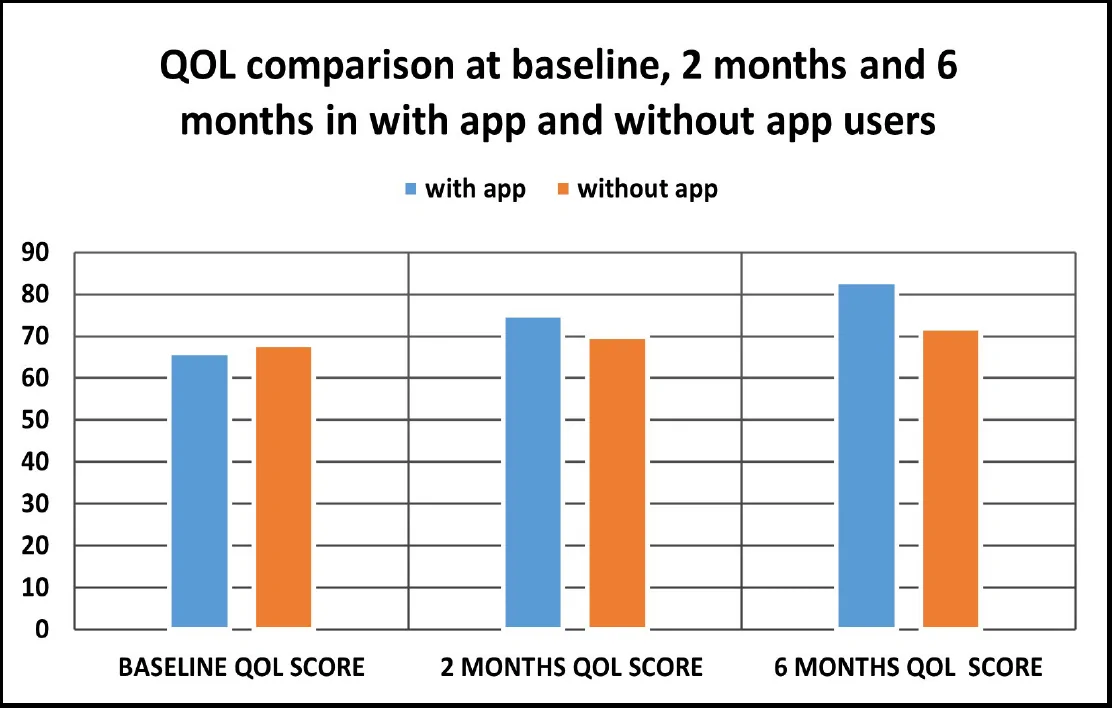
Comparison of QOL Scores at Baseline, 2 Months, and 6 Months Between Mobile App Users and Non-Users.

## DISCUSSION

This study assessed the efficacy of a reminder system based on a mobile application (Medisafe®) in enhancing medication adherence, which in turn improves thyroid function, and symptom management along with better QOL in patients with hypothyroidism. Standard levothyroxine treatment was being administered to these individuals. The findings showed that patients who used the mobile application had significantly better biochemical and symptomatic results than those who did not.

The mobile app users’ mean serum TSH levels at six months were significantly lower than those of non-users suggesting better therapeutic management of thyroid hormone levels. This may be explained by regular medication administration through daily reminders and adherence trackers. This is similar to the findings observed by Kumar et al who reported that inadequate levothyroxine adherence adversely affected thyroid functions, reinforcing the clinical importance of medication adherence and advent of newer technologies to improve long term adherence in people with hypothyroidism.^14^

Mobile Health Applications have become more prevalent in healthcare systems in recent years and have been the focus of several research. With over 4.7 billion smartphone users globally, these apps provide a sustainable, affordable, and widely available approach to controlling chronic illnesses and provides medication adherence.^15^-^17^ In the present study, it was observed that app users reported substantial improvement across nearly all symptoms commonly affected by hypothyroidism. The prevalence of fatigue was significantly reduced among app users compared to non-users, suggesting enhanced energy levels with stable thyroid hormone balance. Similarly, cold intolerance and weight gain, both reflective of impaired metabolism, were considerably lower in the app user group-than in non-users. Improvements were also evident in constipation affecting fewer app users than non-users. Moreover, a notable reduction in excessive sleepiness was observed in app users compared to non-users, reflecting better overall vitality and daily functioning. Menstrual irregularities were significantly less frequent among app users compared to non-users indicating potential stabilization of hormonal rhythms with improved thyroid control. These findings collectively emphasize the critical role of medication adherence in hypothyroidism management. In the same line, a survey-based study by Högqvist Tabor V et al, participants were asked about the utility of the mobile application in supporting their thyroid medication adherence. The findings revealed that those who found the app useful, experienced fewer hypothyroid-related symptoms and reported lower intensity of the remaining symptoms.^18^ Consistent to our findings another study by AlBarrak et al. in people with obesity reported weight reduction and improved overall well-being in people who used the mHealth applications comparative to non-users.^19^

The present results align with current literature emphasizing that forgetfulness and inconsistent dosing are among the primary causes of suboptimal hypothyroidism control,^19^,^20^ and that digital adherence support can mitigate these challenges. The improvement in quality-of-life scores among mobile app users throughout the study period further substantiates the clinical value of technology-assisted adherence strategies which eventually leads towards the better quality of life. By providing personalized reminders and tracking compliance, mobile applications enhance patient engagement and self-management, which are essential in chronic disorders 21,22

A recent study by Mahdavi et al. in pregnant women with hypothyroidism reported that digital health tools and reminder systems enhanced patient self-management and awareness resulting in improved overall health outcomes.^23^ Another study by Zhang et al where an AI based thyroid keeper management system was applied to postoperative thyroid tumor patients enhanced patient doctor communication leading to better health outcomes.^24^ In comparison to these studies which evaluated development, feasibility and usage of these digital tools, our study provides comparative clinical evidence as demonstrated by improved thyroid functions, and quality of life. Moreover, the overall findings suggested that the integration of a mobile app reminder appeared to be a user friendly and cost effective intervention, promoting consistent daily intake of levothyroxine and thus, may be useful in lower and middle income countries (LMIC) where long term medication adherence is challenging.

### Strengths:

To the best of our knowledge, this is the first prospective comparative study from Pakistan to evaluate the use of a mobile health application in the management of patients with hypothyroidism. The study assessed multiple clinically relevant outcomes, including serum TSH levels, hypothyroidism-related symptoms, and quality of life over a six-month follow-up period. The findings demonstrated that the use of a mobile application was associated with improved thyroid function, better symptom control, and enhanced quality of life compared with routine care.

### Limitations:

This was a single-center study with a relatively small sample size. Participants were selected using a non-random consecutive sampling technique, and allocation to the intervention group was based on participant preference, introducing the potential for selection bias. Furthermore, inclusion of only smartphone users may limit the generalizability of the findings. Although levothyroxine dose adjustments were made according to American Thyroid Association (ATA) guidelines, other potential confounding factors, including socioeconomic status, educational level and health literacy were not controlled. In addition, medication adherence was estimated using participant’s self-reported adherence rather than using a validated adherence assessment tool, Self-reported adherence is susceptible to recall and social desirability bias and may not accurately reflect actual medication-taking behavior. Future studies should incorporate objective measures of medication adherence, such as pill counts, pharmacy refill records, or electronic monitoring, and include larger, multicenter randomized controlled studies to validate these findings.

## CONCLUSION

This study demonstrated that the use of a mobile application significantly improved treatment adherence, normalized thyroid stimulating hormone levels, and alleviation of classical hypothyroid-related symptoms with improved quality of life. Moreover, the findings suggest that integrating mobile-based reminder tools into routine hypothyroidism management could optimize therapeutic outcomes, particularly in patients struggling with daily medication adherence. Future multicenter studies with larger sample sizes and extended follow-up periods are recommended to validate these findings and explore additional benefits of digital health interventions in chronic disease management.
